# (1R*,21S*,22R*,24S*)-Methyl ethyl 2-[23-hy­droxy-22,24-diphenyl-8,11,14-trioxa-25-aza­tetra­cyclo­[19.3.1.0^2,7^.0^15,20^]penta­cosa-2,4,6,15(20),16,18-hexaen-25-yl]but-2-enedioate

**DOI:** 10.1107/S1600536813014748

**Published:** 2013-06-08

**Authors:** Truong Hong Hieu, Le Tuan Anh, Anatoly T. Soldatenkov, Olga S. Gorchakova, Victor N. Khrustalev

**Affiliations:** a Department of Chemistry, Vietnam National University, 144 Xuan Thuy, Cau Giay, Hanoi, Vietnam; b Organic Chemistry Department, Russian Peoples Friendship University, Miklukho-Maklaya St. 6, Moscow, 117198, Russian Federation; c X-Ray Structural Centre, A.N. Nesmeyanov Institute of Organoelement Compounds, Russian Academy of Sciences, 28 Vavilov St., B-334, Moscow 119991, Russian Federation

## Abstract

The title compound, C_40_H_41_NO_8_, is a product of the reduction of the cyclic carbonyl group of the γ-piperidone subunit of the aza-14-crown-4 ether with subsequent re-esterification of its dimethyl butenoate substituent into a monoethyl monomethyl group. The aza­crown macrocycle exhibits a bowl conformation with a dihedral angle of 70.82 (5)° between the benzene rings fused to it. The piperidine ring adopts a chair conformation and the methyl ethyl ethyl­enedi­carboxyl­ate fragment has a *cis* conformation, with a dihedral angle of 66.51 (7)° between the two carboxyl­ate groups. The ethyl group is disordered over two sites with occupancies of 0.70 (1):0.30 (1). In the crystal, mol­ecules form inversion dimers, *via* pairs of O—H⋯O hydrogen bonds, that stack along the *a* axis.

## Related literature

For the synthesis of aza­crown ethers of this type, see: Levov *et al.* (2006 ▶, 2008 ▶); Anh *et al.* (2008 ▶); Hieu *et al.* (2011 ▶); Khieu *et al.* (2011 ▶). For the structures of related compounds, see: Anh *et al.* (2012*a* ▶,*b* ▶); Hieu *et al.* (2012 ▶).
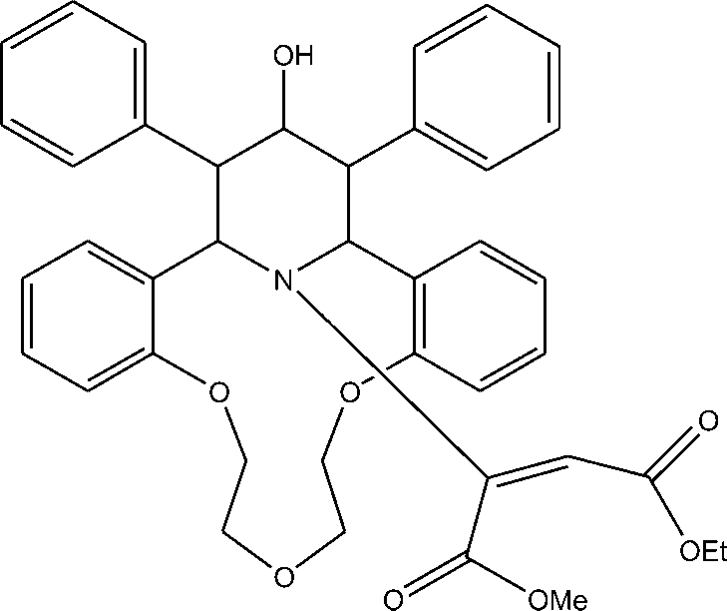


## Experimental

### 

#### Crystal data

C_40_H_41_NO_8_
*M* *_r_* = 663.74
Monoclinic, 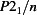

*a* = 11.6594 (4) Å
*b* = 19.3088 (6) Å
*c* = 15.8522 (5) Å
β = 108.887 (1)°
*V* = 3376.64 (19) Å^3^
*Z* = 4
Mo *K*α radiation
μ = 0.09 mm^−1^
*T* = 100 K
0.18 × 0.15 × 0.12 mm

#### Data collection

Bruker APEXII CCD diffractometer
Absorption correction: multi-scan (*SADABS*; Bruker, 2003 ▶) *T*_min_ = 0.984, *T*_max_ = 0.989
44045 measured reflections
9846 independent reflections
6852 reflections with *I* > 2σ(*I*)
*R*_int_ = 0.041

#### Refinement

*R*[*F* ^2^ > 2σ(*F* ^2^)] = 0.048
*wR*(*F* ^2^) = 0.136
*S* = 1.00
9846 reflections
452 parameters
4 restraints
H atoms treated by a mixture of independent and constrained refinement
Δρ_max_ = 0.42 e Å^−3^
Δρ_min_ = −0.48 e Å^−3^

### 

Data collection: *APEX2* (Bruker, 2005 ▶); cell refinement: *SAINT* (Bruker, 2001 ▶); data reduction: *SAINT*; program(s) used to solve structure: *SHELXTL* (Sheldrick, 2008 ▶); program(s) used to refine structure: *SHELXTL*; molecular graphics: *SHELXTL*; software used to prepare material for publication: *SHELXTL*.

## Supplementary Material

Crystal structure: contains datablock(s) global, I. DOI: 10.1107/S1600536813014748/rk2405sup1.cif

Structure factors: contains datablock(s) I. DOI: 10.1107/S1600536813014748/rk2405Isup2.hkl

Supplementary material file. DOI: 10.1107/S1600536813014748/rk2405Isup3.cml

Additional supplementary materials:  crystallographic information; 3D view; checkCIF report

## Figures and Tables

**Table 1:** Hydrogen-bond geometry (Å, °)

*D*—H⋯*A*	*D*—H	H⋯*A*	*D*⋯*A*	*D*—H⋯*A*
O23—H23*O*⋯O43^i^	0.82 (2)	2.39 (2)	3.1109 (14)	148 (2)

Symmetry code: (i) 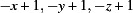.

**Fig. 1.**
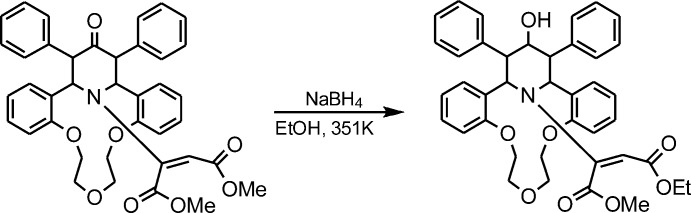
The reaction of reduction and subsequent re-esterification of initial dimethyl 2-[bis(benzo)-(β,β'-diphenyl-γ-piperidono)aza-14-crown-4-ether]butenoate.

**Fig. 2.**
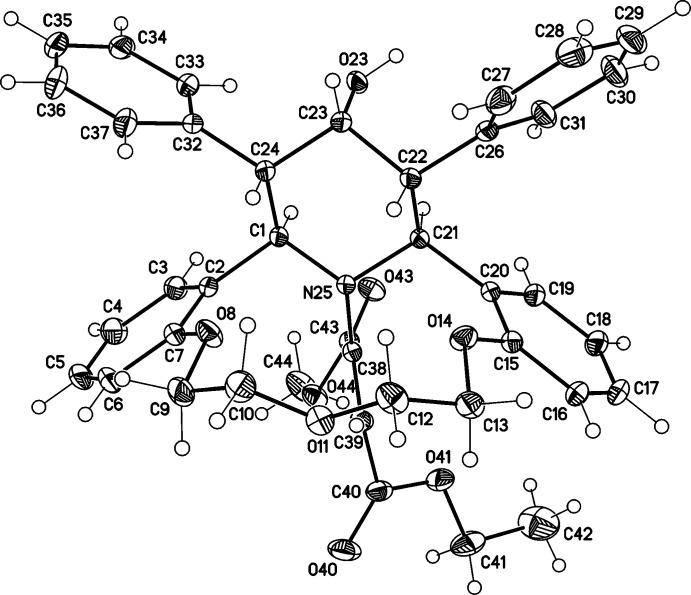
Molecular structure of I. Displacement ellipsoids are shown at the 50% probability level. H atoms are presented as small spheres of arbitraryradius. The alternative minor position of the disordered ethyl group is not depicted.

**Fig. 3.**
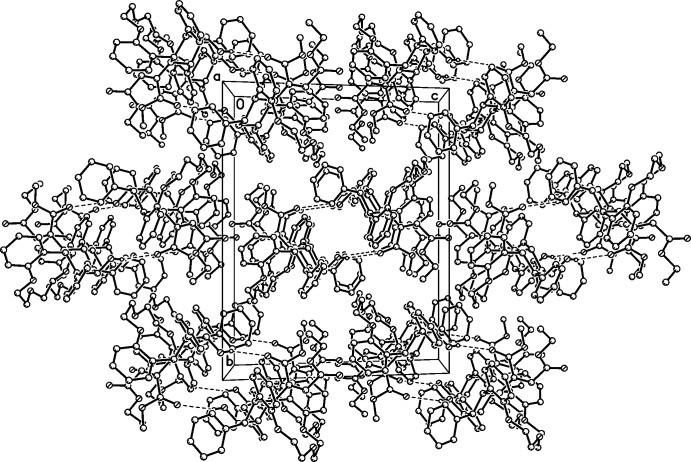
The projection of the crystal structure of I along the a axis demonstrating the packing of the centrosymmetrical dimers. Dashed lines indicate the intermolecular O–H···O hydrogen bonds.

## supplementary crystallographic information

### Comment

Recently we have developed effective methods of synthesis of azacrown ethers including piperidine (Levov *et al.*, 2006, 2008; Anh *et al.*, 2008), perhydropyrimidine (Hieu *et al.*, 2011) and perhydrotriazine subunits (Khieu *et al.*, 2011). Currently we study their structures and properties systematically (Anh *et al.*, 2012*a*,*b*; Hieu *et al.*, 2012). In attempt to reduce the cyclic carbonyl group of the γ-piperidone subunit into the carbinol one of the initial bis(benzo)-(β,β'-diphenyl-γ-piperidono)aza-14-crown-4 ether containing *N*-(dimethyl)maleinate fragment, we found that the expected reduction was accompanied by re-esterification of one methoxy group of the dimethyl butenoate substituent into the ethoxy one (Fig. 1). The structure of the resulting compound - the higher sterically hindered product (I) was unambiguously established by X-ray diffraction analysis.

The title compound I, C_40_H_41_NO_8_, comprises the aza-14-crown-4-ether skeletal moiety and adopts a bowl conformation (Fig. 2). The configuration of the C7-O8-C9-C10-O11-C12-C13-O14-C15 polyether chain is t-g^(-)^-t-t-g^(+)^-t (t = *trans*, 180°; g = *gauche*, ±60°). The piperidine ring of the bicyclic fragment have a *chair* conformation. The dihedral angle between the planes of the benzene rings fused to the aza-14-crown-4-ether moiety is 70.82 (5)°. The phenyl rings at the C22 and C24 carbon atoms occupy the sterically favorable equatorial positions, and are rotated to each other by 65.00 (6)°. Contrary to that, the hydroxyl group at the C23 carbon atom occupies the axial position. The methyl ethyl ethylenedicarboxylate fragment has a *cis* configuration with the dihedral angle of 66.51 (7)° between the two carboxylate groups. The ethyl group is disordered over two sites with the occupancies of 0.70 (1):0.30 (1). The volume of the internal cavity of macrocycle I is approximately equal to 61Å^3^.

The molecule of I possesses four asymmetric centers at the C1, C21, C22 and C24 carbon atoms and can have potentially numerous diastereomers. The crystal of I is racemic and consists of enantiomeric pairs with the following relative configuration of the centers: *rac*-1*R**,21*S**,22*R**,24*S**.

In the crystal, the molecules of I form centrosymmetrical dimers by the intermolecular O23–H23···O43^i^ hydrogen bonds (Fig. 3, Table 1). The crystal packing of the dimers is stacking along the *a* axis (Fig. 3). Symmetry code: (i) -*x*+1, -*y*+1, -*z*+1.

### Experimental

A powder of NaBH_4_ (1.14 g, 30 mmol) was added to a suspension of azacrown ether (6.47 g, 10 mmol) in ethanol (50 ml). The mixture was stirred for 30 min at r.t. and then boiled for 1 h. After the solvent evaporation, the residue was washed with hot water (30 ml) and purified by re-crystallization from ethanol to give 2.27 g of colourless crystals of I. Yield is 34%. M.p. = 526-528 K. IR (KBr), ν/cm^-1^: 3436, 1714. ^1^H NMR (CDCl_3_, 400 MHz, 300 K): δ = 0,82 (t, 3H, ^3^*J* = 5.5, OCH_2_C*H*_3_), 3.48 (s, 3H, OCH_3_), 3.67 (q, 2H, ^3^*J* = 5.5, C*H*_2_CH_3_), 3.85-4.20 (m, 11H, 2×OCH_2_CH_2_O and H22, H23, H24), 4.37 (d, 2H, ^3^*J* = 10.5, H1, H21), 5.02 (s, 1H, OH), 6.51 (m, 3H, H_arom_), 6.63 (s, 1H, C═CHCOO), 6.45-6.67 (m, 3H, H_arom_), 6.88-7.15 (m, 12H, H_arom_). Mass-spectrum (LCMS), m/*z*: 664 [*M*+1]^+^. Anal. Calcd. for C_40_H_41_NO_8_: C, 72.38; H, 6.23; N 2.11. Found: C, 72.32; H, 6.19; N, 2.08.

### Refinement

The 4 distance restraints were used to fit the ideal conformations for both orientations of the disordered ethyl group. The C–C distances were fixed at 1.500 (3)Å (C41–C42, C41–C42') (two restraints). The corresponding O···C distances (O41···C42, O41···C42') were fixed at 2.420 (3)Å (two restraints). Moreover, it was taken into account that the anisotropic displacement parameters for the C42 and C42' carbon atoms of the ethyl group are equal (one restraint).

The hydrogen atoms were placed in calculated positions with C–H = 0.95-1.00Å and refined in the riding model with fixed isotropic displacement parameters [*U*_iso_(H) = 1.5*U*_eq_(C) for the methyl groups and *U*_iso_(H) = 1.2*U*_eq_(C) for the other groups].

### Crystal data

**Table:**

C_40_H_41_NO_8_	*F*(000) = 1408
*M**_r_* = 663.74	*D*_x_ = 1.306 Mg m^−^^3^
Monoclinic, *P*2_1_/*n*	Melting point = 526–528 K
Hall symbol: -P 2yn	Mo *K*α radiation, λ = 0.71073 Å
*a* = 11.6594 (4) Å	Cell parameters from 9431 reflections
*b* = 19.3088 (6) Å	θ = 2.5–30.4°
*c* = 15.8522 (5) Å	µ = 0.09 mm^−^^1^
β = 108.887 (1)°	*T* = 100 K
*V* = 3376.64 (19) Å^3^	Prism, colourless
*Z* = 4	0.18 × 0.15 × 0.12 mm

### Data collection

**Table:**

Bruker APEXII CCD diffractometer	9846 independent reflections
Radiation source: fine-focus sealed tube	6852 reflections with *I* > 2σ(*I*)
Graphite monochromator	*R*_int_ = 0.041
φ and ω scans	θ_max_ = 30.0°, θ_min_ = 1.7°
Absorption correction: multi-scan (*SADABS*; Bruker, 2003)	*h* = −16→16
*T*_min_ = 0.984, *T*_max_ = 0.989	*k* = −27→27
44045 measured reflections	*l* = −22→22

### Refinement

**Table:**

Refinement on *F*^2^	Primary atom site location: structure-invariant direct methods
Least-squares matrix: full	Secondary atom site location: difference Fourier map
*R*[*F*^2^ > 2σ(*F*^2^)] = 0.048	Hydrogen site location: difference Fourier map
*wR*(*F*^2^) = 0.136	H atoms treated by a mixture of independent and constrained refinement
*S* = 1.00	*w* = 1/[σ^2^(*F*_o_^2^) + (0.0675*P*)^2^ + 0.867*P*] where *P* = (*F*_o_^2^ + 2*F*_c_^2^)/3
9846 reflections	(Δ/σ)_max_ < 0.001
452 parameters	Δρ_max_ = 0.42 e Å^−^^3^
4 restraints	Δρ_min_ = −0.48 e Å^−^^3^

### Special details

**Table:**

Geometry. All s.u.'s (except the s.u. in the dihedral angle between two l.s. planes) are estimated using the full covariance matrix. The cell s.u.'s are taken into account individually in the estimation of s.u.'s in distances, angles and torsion angles; correlations between s.u.'s in cell parameters are only used when they are defined by crystal symmetry. An approximate (isotropic) treatment of cell s.u.'s is used for estimating s.u.'s involving l.s. planes.
Refinement. Refinement of *F*^2^ against ALL reflections. The weighted *R*-factor *wR* and goodness of fit *S* are based on *F*^2^, conventional *R*-factors *R* are based on *F*, with *F* set to zero for negative *F*^2^. The threshold expression of *F*^2^ > σ(*F*^2^) is used only for calculating *R*-factors(gt) *etc*. and is not relevant to the choice of reflections for refinement. *R*-factors based on *F*^2^ are statistically about twice as large as those based on *F*, and *R*-factors based on ALL data will be even larger.

### Fractional atomic coordinates and isotropic or equivalent isotropic displacement parameters (Å^2^)

**Table:**

	*x*	*y*	*z*	*U*_iso_*/*U*_eq_	Occ. (<1)
C1	0.49983 (11)	0.57458 (7)	0.29788 (8)	0.0224 (2)	
H1	0.5405	0.5351	0.3371	0.027*	
C2	0.53553 (12)	0.57269 (7)	0.21364 (9)	0.0252 (3)	
C3	0.61542 (13)	0.52223 (9)	0.20458 (10)	0.0342 (3)	
H3	0.6468	0.4898	0.2514	0.041*	
C4	0.65123 (15)	0.51760 (11)	0.12888 (12)	0.0442 (4)	
H4	0.7066	0.4827	0.1247	0.053*	
C5	0.60556 (16)	0.56398 (11)	0.06046 (11)	0.0459 (4)	
H5	0.6289	0.5610	0.0085	0.055*	
C6	0.52562 (15)	0.61508 (10)	0.06706 (10)	0.0404 (4)	
H6	0.4945	0.6470	0.0196	0.049*	
C7	0.49050 (13)	0.61998 (8)	0.14288 (9)	0.0298 (3)	
O8	0.41266 (10)	0.66954 (6)	0.15329 (7)	0.0355 (2)	
C9	0.35409 (15)	0.71378 (10)	0.07948 (11)	0.0403 (4)	
H9A	0.3138	0.6859	0.0256	0.048*	
H9B	0.4141	0.7447	0.0665	0.048*	
C10	0.26254 (15)	0.75544 (9)	0.10525 (12)	0.0404 (4)	
H10A	0.3006	0.7767	0.1646	0.048*	
H10B	0.2304	0.7929	0.0614	0.048*	
O11	0.16716 (10)	0.71066 (6)	0.10756 (7)	0.0379 (3)	
C12	0.09276 (16)	0.73823 (9)	0.15415 (11)	0.0388 (4)	
H12A	0.0314	0.7696	0.1147	0.047*	
H12B	0.1426	0.7651	0.2063	0.047*	
C13	0.03124 (14)	0.67958 (9)	0.18435 (10)	0.0344 (3)	
H13A	−0.0326	0.6975	0.2072	0.041*	
H13B	−0.0066	0.6477	0.1341	0.041*	
O14	0.12256 (9)	0.64436 (5)	0.25342 (7)	0.0313 (2)	
C15	0.09638 (12)	0.58011 (7)	0.27916 (9)	0.0258 (3)	
C16	−0.02065 (13)	0.55346 (9)	0.25550 (10)	0.0333 (3)	
H16	−0.0872	0.5801	0.2198	0.040*	
C17	−0.03998 (14)	0.48800 (10)	0.28412 (11)	0.0393 (4)	
H17	−0.1200	0.4700	0.2681	0.047*	
C18	0.05563 (14)	0.44881 (9)	0.33557 (11)	0.0366 (3)	
H18	0.0419	0.4041	0.3554	0.044*	
C19	0.17243 (13)	0.47531 (8)	0.35823 (9)	0.0282 (3)	
H19	0.2383	0.4479	0.3932	0.034*	
C20	0.19565 (12)	0.54073 (7)	0.33120 (8)	0.0231 (3)	
C21	0.32667 (11)	0.56407 (7)	0.35540 (8)	0.0208 (2)	
H21	0.3779	0.5290	0.3974	0.025*	
C22	0.35632 (12)	0.63557 (7)	0.40031 (8)	0.0221 (2)	
H22	0.3200	0.6712	0.3534	0.027*	
C23	0.49512 (12)	0.64603 (7)	0.43145 (8)	0.0232 (3)	
H23	0.5143	0.6929	0.4594	0.028*	
O23	0.55753 (9)	0.59506 (6)	0.49501 (6)	0.0281 (2)	
H23O	0.5347 (18)	0.5986 (10)	0.5383 (14)	0.045 (5)*	
C24	0.53900 (12)	0.64288 (7)	0.35017 (8)	0.0233 (3)	
H24	0.4960	0.6809	0.3094	0.028*	
N25	0.36706 (9)	0.56630 (6)	0.27489 (7)	0.0216 (2)	
C26	0.30571 (12)	0.64843 (8)	0.47576 (9)	0.0271 (3)	
C27	0.27685 (14)	0.71585 (9)	0.49246 (12)	0.0411 (4)	
H27	0.2883	0.7525	0.4560	0.049*	
C28	0.23144 (16)	0.73048 (13)	0.56181 (14)	0.0586 (6)	
H28	0.2143	0.7770	0.5734	0.070*	
C29	0.21149 (18)	0.67744 (15)	0.61346 (13)	0.0632 (7)	
H29	0.1788	0.6871	0.6599	0.076*	
C30	0.23885 (18)	0.61087 (14)	0.59774 (12)	0.0576 (6)	
H30	0.2242	0.5743	0.6330	0.069*	
C31	0.28807 (15)	0.59595 (10)	0.53049 (10)	0.0387 (4)	
H31	0.3097	0.5497	0.5220	0.046*	
C32	0.67345 (12)	0.65566 (8)	0.36966 (9)	0.0271 (3)	
C33	0.76284 (13)	0.61124 (9)	0.42065 (10)	0.0328 (3)	
H33	0.7403	0.5717	0.4472	0.039*	
C34	0.88485 (14)	0.62371 (10)	0.43350 (11)	0.0418 (4)	
H34	0.9446	0.5927	0.4686	0.050*	
C35	0.91940 (16)	0.68080 (12)	0.39551 (12)	0.0501 (5)	
H35	1.0027	0.6896	0.4046	0.060*	
C36	0.83257 (17)	0.72469 (11)	0.34460 (14)	0.0534 (5)	
H36	0.8558	0.7640	0.3179	0.064*	
C37	0.71051 (15)	0.71244 (9)	0.33146 (12)	0.0410 (4)	
H37	0.6514	0.7435	0.2957	0.049*	
C38	0.31867 (12)	0.51263 (7)	0.21213 (8)	0.0228 (2)	
C39	0.22377 (12)	0.52554 (8)	0.14012 (9)	0.0277 (3)	
H39	0.1989	0.5724	0.1289	0.033*	
C40	0.15353 (13)	0.47283 (9)	0.07592 (10)	0.0331 (3)	
O40	0.13153 (11)	0.47494 (7)	−0.00366 (7)	0.0477 (3)	
O41	0.11288 (10)	0.42495 (6)	0.11879 (8)	0.0414 (3)	
C41	0.02979 (17)	0.37424 (10)	0.06340 (12)	0.0507 (5)	
H41A	0.0732	0.3440	0.0333	0.061*	0.70
H41B	−0.0366	0.3980	0.0171	0.061*	0.70
H41C	0.0753	0.3329	0.0553	0.061*	0.30
H41D	−0.0125	0.3943	0.0039	0.061*	0.30
C42	−0.0209 (4)	0.33185 (17)	0.1222 (2)	0.0801 (13)	0.70
H42A	0.0446	0.3055	0.1645	0.120*	0.70
H42B	−0.0820	0.2998	0.0855	0.120*	0.70
H42C	−0.0584	0.3625	0.1550	0.120*	0.70
C42'	−0.0642 (6)	0.3559 (5)	0.1058 (5)	0.0801 (13)	0.30
H42D	−0.1233	0.3936	0.0962	0.120*	0.30
H42E	−0.0249	0.3488	0.1699	0.120*	0.30
H42F	−0.1057	0.3132	0.0790	0.120*	0.30
C43	0.37713 (12)	0.44258 (7)	0.23154 (9)	0.0253 (3)	
O43	0.42283 (11)	0.41895 (6)	0.30529 (7)	0.0355 (2)	
O44	0.37842 (10)	0.41068 (5)	0.15658 (7)	0.0331 (2)	
C44	0.4458 (2)	0.34731 (10)	0.16785 (13)	0.0523 (5)	
H44A	0.4741	0.3403	0.1166	0.078*	
H44B	0.3937	0.3085	0.1721	0.078*	
H44C	0.5157	0.3498	0.2225	0.078*	

### Atomic displacement parameters (Å^2^)

**Table:**

	*U*^11^	*U*^22^	*U*^33^	*U*^12^	*U*^13^	*U*^23^
C1	0.0179 (5)	0.0297 (6)	0.0198 (6)	−0.0009 (5)	0.0065 (5)	0.0019 (5)
C2	0.0191 (6)	0.0362 (7)	0.0211 (6)	−0.0037 (5)	0.0075 (5)	−0.0021 (5)
C3	0.0250 (7)	0.0477 (9)	0.0305 (7)	0.0035 (6)	0.0100 (6)	−0.0024 (6)
C4	0.0306 (8)	0.0663 (12)	0.0391 (9)	0.0067 (8)	0.0162 (7)	−0.0098 (8)
C5	0.0351 (8)	0.0795 (13)	0.0285 (8)	−0.0013 (8)	0.0178 (7)	−0.0057 (8)
C6	0.0368 (8)	0.0633 (11)	0.0249 (7)	−0.0022 (8)	0.0150 (6)	0.0038 (7)
C7	0.0258 (7)	0.0423 (8)	0.0225 (6)	−0.0036 (6)	0.0093 (5)	0.0006 (6)
O8	0.0399 (6)	0.0437 (6)	0.0248 (5)	0.0099 (5)	0.0131 (4)	0.0109 (4)
C9	0.0382 (8)	0.0535 (10)	0.0289 (8)	0.0028 (7)	0.0103 (6)	0.0185 (7)
C10	0.0393 (9)	0.0396 (9)	0.0391 (9)	0.0022 (7)	0.0084 (7)	0.0158 (7)
O11	0.0379 (6)	0.0427 (6)	0.0349 (6)	−0.0014 (5)	0.0141 (5)	0.0001 (5)
C12	0.0434 (9)	0.0385 (8)	0.0346 (8)	0.0119 (7)	0.0128 (7)	0.0074 (7)
C13	0.0283 (7)	0.0439 (9)	0.0287 (7)	0.0120 (6)	0.0062 (6)	0.0068 (6)
O14	0.0245 (5)	0.0342 (5)	0.0304 (5)	0.0024 (4)	0.0022 (4)	0.0063 (4)
C15	0.0219 (6)	0.0337 (7)	0.0225 (6)	0.0005 (5)	0.0079 (5)	−0.0014 (5)
C16	0.0194 (6)	0.0489 (9)	0.0306 (7)	0.0014 (6)	0.0066 (5)	0.0007 (6)
C17	0.0218 (7)	0.0586 (10)	0.0371 (8)	−0.0110 (7)	0.0089 (6)	0.0012 (7)
C18	0.0312 (8)	0.0433 (9)	0.0349 (8)	−0.0113 (6)	0.0104 (6)	0.0044 (7)
C19	0.0244 (6)	0.0345 (7)	0.0259 (6)	−0.0030 (5)	0.0084 (5)	0.0030 (5)
C20	0.0199 (6)	0.0313 (7)	0.0189 (6)	−0.0015 (5)	0.0075 (5)	−0.0013 (5)
C21	0.0191 (6)	0.0260 (6)	0.0174 (5)	0.0001 (5)	0.0062 (4)	0.0012 (5)
C22	0.0206 (6)	0.0260 (6)	0.0194 (6)	0.0007 (5)	0.0060 (5)	0.0008 (5)
C23	0.0211 (6)	0.0277 (6)	0.0200 (6)	−0.0023 (5)	0.0056 (5)	0.0011 (5)
O23	0.0226 (5)	0.0426 (6)	0.0190 (4)	0.0026 (4)	0.0068 (4)	0.0057 (4)
C24	0.0206 (6)	0.0294 (6)	0.0198 (6)	−0.0033 (5)	0.0063 (5)	0.0016 (5)
N25	0.0179 (5)	0.0291 (6)	0.0183 (5)	−0.0019 (4)	0.0064 (4)	−0.0014 (4)
C26	0.0181 (6)	0.0390 (8)	0.0226 (6)	−0.0005 (5)	0.0046 (5)	−0.0060 (5)
C27	0.0292 (8)	0.0450 (9)	0.0487 (9)	0.0000 (7)	0.0121 (7)	−0.0143 (8)
C28	0.0341 (9)	0.0770 (14)	0.0624 (13)	0.0076 (9)	0.0125 (9)	−0.0398 (11)
C29	0.0378 (10)	0.120 (2)	0.0341 (9)	0.0092 (11)	0.0150 (8)	−0.0224 (11)
C30	0.0445 (10)	0.1052 (18)	0.0279 (8)	0.0089 (11)	0.0183 (8)	0.0054 (10)
C31	0.0378 (8)	0.0556 (10)	0.0256 (7)	0.0054 (7)	0.0141 (6)	0.0045 (7)
C32	0.0242 (6)	0.0374 (7)	0.0201 (6)	−0.0084 (5)	0.0077 (5)	−0.0040 (5)
C33	0.0238 (7)	0.0462 (9)	0.0284 (7)	−0.0037 (6)	0.0085 (6)	−0.0019 (6)
C34	0.0243 (7)	0.0674 (12)	0.0326 (8)	−0.0023 (7)	0.0079 (6)	−0.0099 (8)
C35	0.0290 (8)	0.0812 (14)	0.0439 (10)	−0.0225 (9)	0.0169 (7)	−0.0183 (9)
C36	0.0415 (10)	0.0658 (13)	0.0567 (11)	−0.0256 (9)	0.0212 (9)	0.0018 (10)
C37	0.0351 (8)	0.0476 (10)	0.0412 (9)	−0.0124 (7)	0.0135 (7)	0.0057 (7)
C38	0.0211 (6)	0.0288 (6)	0.0201 (6)	−0.0008 (5)	0.0087 (5)	−0.0018 (5)
C39	0.0243 (6)	0.0361 (7)	0.0222 (6)	0.0027 (5)	0.0068 (5)	−0.0035 (5)
C40	0.0239 (7)	0.0449 (9)	0.0267 (7)	0.0066 (6)	0.0029 (5)	−0.0089 (6)
O40	0.0460 (7)	0.0662 (8)	0.0248 (6)	0.0053 (6)	0.0029 (5)	−0.0115 (5)
O41	0.0369 (6)	0.0493 (7)	0.0343 (6)	−0.0101 (5)	0.0064 (5)	−0.0150 (5)
C41	0.0451 (10)	0.0455 (10)	0.0510 (11)	−0.0062 (8)	0.0007 (8)	−0.0205 (8)
C42	0.105 (3)	0.058 (2)	0.069 (2)	−0.025 (2)	0.018 (2)	−0.0062 (18)
C42'	0.105 (3)	0.058 (2)	0.069 (2)	−0.025 (2)	0.018 (2)	−0.0062 (18)
C43	0.0236 (6)	0.0290 (7)	0.0240 (6)	−0.0014 (5)	0.0088 (5)	−0.0024 (5)
O43	0.0450 (6)	0.0329 (6)	0.0271 (5)	0.0062 (5)	0.0096 (5)	0.0039 (4)
O44	0.0392 (6)	0.0345 (6)	0.0277 (5)	0.0094 (5)	0.0137 (4)	−0.0024 (4)
C44	0.0783 (14)	0.0416 (9)	0.0440 (10)	0.0261 (9)	0.0296 (10)	0.0034 (8)

### Geometric parameters (Å, º)

**Table:**

C1—N25	1.4797 (16)	C24—C32	1.5166 (18)
C1—C2	1.5225 (17)	C24—H24	1.0000
C1—C24	1.5459 (18)	N25—C38	1.4208 (17)
C1—H1	1.0000	C26—C27	1.391 (2)
C2—C3	1.387 (2)	C26—C31	1.392 (2)
C2—C7	1.409 (2)	C27—C28	1.395 (3)
C3—C4	1.395 (2)	C27—H27	0.9500
C3—H3	0.9500	C28—C29	1.377 (3)
C4—C5	1.374 (3)	C28—H28	0.9500
C4—H4	0.9500	C29—C30	1.367 (3)
C5—C6	1.384 (3)	C29—H29	0.9500
C5—H5	0.9500	C30—C31	1.395 (2)
C6—C7	1.3928 (19)	C30—H30	0.9500
C6—H6	0.9500	C31—H31	0.9500
C7—O8	1.3651 (19)	C32—C37	1.387 (2)
O8—C9	1.4310 (18)	C32—C33	1.389 (2)
C9—C10	1.495 (2)	C33—C34	1.391 (2)
C9—H9A	0.9900	C33—H33	0.9500
C9—H9B	0.9900	C34—C35	1.377 (3)
C10—O11	1.419 (2)	C34—H34	0.9500
C10—H10A	0.9900	C35—C36	1.366 (3)
C10—H10B	0.9900	C35—H35	0.9500
O11—C12	1.4127 (19)	C36—C37	1.390 (2)
C12—C13	1.500 (2)	C36—H36	0.9500
C12—H12A	0.9900	C37—H37	0.9500
C12—H12B	0.9900	C38—C39	1.3311 (19)
C13—O14	1.4283 (17)	C38—C43	1.5010 (19)
C13—H13A	0.9900	C39—C40	1.484 (2)
C13—H13B	0.9900	C39—H39	0.9500
O14—C15	1.3710 (17)	C40—O40	1.2040 (18)
C15—C16	1.3914 (19)	C40—O41	1.323 (2)
C15—C20	1.4071 (19)	O41—C41	1.455 (3)
C16—C17	1.386 (2)	C41—C42	1.498 (3)
C16—H16	0.9500	C41—C42'	1.501 (3)
C17—C18	1.375 (2)	C41—H41A	0.9900
C17—H17	0.9500	C41—H41B	0.9900
C18—C19	1.389 (2)	C41—H41C	0.9901
C18—H18	0.9500	C41—H41D	0.9901
C19—C20	1.3885 (19)	C42—H42A	0.9800
C19—H19	0.9500	C42—H42B	0.9800
C20—C21	1.5180 (17)	C42—H42C	0.9800
C21—N25	1.4972 (15)	C42'—H42D	0.9800
C21—C22	1.5405 (18)	C42'—H42E	0.9800
C21—H21	1.0000	C42'—H42F	0.9800
C22—C26	1.5157 (18)	C43—O43	1.2065 (17)
C22—C23	1.5448 (18)	C43—O44	1.3429 (16)
C22—H22	1.0000	O44—C44	1.4337 (19)
C23—O23	1.4278 (16)	C44—H44A	0.9800
C23—C24	1.5337 (17)	C44—H44B	0.9800
C23—H23	1.0000	C44—H44C	0.9800
O23—H23O	0.81 (2)		
			
N25—C1—C2	110.04 (10)	C23—O23—H23O	107.8 (14)
N25—C1—C24	109.16 (10)	C32—C24—C23	115.23 (11)
C2—C1—C24	111.93 (10)	C32—C24—C1	110.76 (11)
N25—C1—H1	108.5	C23—C24—C1	111.30 (10)
C2—C1—H1	108.5	C32—C24—H24	106.3
C24—C1—H1	108.5	C23—C24—H24	106.3
C3—C2—C7	117.54 (13)	C1—C24—H24	106.3
C3—C2—C1	119.41 (13)	C38—N25—C1	113.45 (10)
C7—C2—C1	123.05 (12)	C38—N25—C21	114.38 (10)
C2—C3—C4	122.13 (15)	C1—N25—C21	112.69 (10)
C2—C3—H3	118.9	C27—C26—C31	117.97 (14)
C4—C3—H3	118.9	C27—C26—C22	118.90 (14)
C5—C4—C3	119.32 (16)	C31—C26—C22	123.12 (13)
C5—C4—H4	120.3	C26—C27—C28	121.09 (19)
C3—C4—H4	120.3	C26—C27—H27	119.5
C4—C5—C6	120.22 (14)	C28—C27—H27	119.5
C4—C5—H5	119.9	C29—C28—C27	119.88 (19)
C6—C5—H5	119.9	C29—C28—H28	120.1
C5—C6—C7	120.45 (16)	C27—C28—H28	120.1
C5—C6—H6	119.8	C30—C29—C28	119.79 (17)
C7—C6—H6	119.8	C30—C29—H29	120.1
O8—C7—C6	123.00 (14)	C28—C29—H29	120.1
O8—C7—C2	116.67 (12)	C29—C30—C31	120.8 (2)
C6—C7—C2	120.33 (14)	C29—C30—H30	119.6
C7—O8—C9	118.65 (12)	C31—C30—H30	119.6
O8—C9—C10	106.96 (12)	C26—C31—C30	120.43 (18)
O8—C9—H9A	110.3	C26—C31—H31	119.8
C10—C9—H9A	110.3	C30—C31—H31	119.8
O8—C9—H9B	110.3	C37—C32—C33	117.44 (14)
C10—C9—H9B	110.3	C37—C32—C24	119.15 (14)
H9A—C9—H9B	108.6	C33—C32—C24	123.33 (13)
O11—C10—C9	108.30 (14)	C32—C33—C34	121.15 (15)
O11—C10—H10A	110.0	C32—C33—H33	119.4
C9—C10—H10A	110.0	C34—C33—H33	119.4
O11—C10—H10B	110.0	C35—C34—C33	120.29 (17)
C9—C10—H10B	110.0	C35—C34—H34	119.9
H10A—C10—H10B	108.4	C33—C34—H34	119.9
C12—O11—C10	113.76 (13)	C36—C35—C34	119.29 (15)
O11—C12—C13	108.69 (13)	C36—C35—H35	120.4
O11—C12—H12A	110.0	C34—C35—H35	120.4
C13—C12—H12A	110.0	C35—C36—C37	120.65 (17)
O11—C12—H12B	110.0	C35—C36—H36	119.7
C13—C12—H12B	110.0	C37—C36—H36	119.7
H12A—C12—H12B	108.3	C32—C37—C36	121.17 (17)
O14—C13—C12	106.82 (13)	C32—C37—H37	119.4
O14—C13—H13A	110.4	C36—C37—H37	119.4
C12—C13—H13A	110.4	C39—C38—N25	119.70 (12)
O14—C13—H13B	110.4	C39—C38—C43	122.73 (12)
C12—C13—H13B	110.4	N25—C38—C43	117.56 (11)
H13A—C13—H13B	108.6	C38—C39—C40	125.50 (14)
C15—O14—C13	118.45 (11)	C38—C39—H39	117.3
O14—C15—C16	123.21 (13)	C40—C39—H39	117.3
O14—C15—C20	116.32 (12)	O40—C40—O41	124.87 (15)
C16—C15—C20	120.47 (13)	O40—C40—C39	125.34 (16)
C17—C16—C15	119.88 (14)	O41—C40—C39	109.69 (12)
C17—C16—H16	120.1	C40—O41—C41	116.08 (12)
C15—C16—H16	120.1	O41—C41—C42	108.18 (15)
C18—C17—C16	120.63 (14)	O41—C41—C42'	109.19 (19)
C18—C17—H17	119.7	O41—C41—H41A	110.1
C16—C17—H17	119.7	C42—C41—H41A	110.1
C17—C18—C19	119.29 (15)	O41—C41—H41B	110.1
C17—C18—H18	120.4	C42—C41—H41B	110.1
C19—C18—H18	120.4	H41A—C41—H41B	108.4
C20—C19—C18	121.89 (14)	O41—C41—H41C	109.9
C20—C19—H19	119.1	C42'—C41—H41C	111.5
C18—C19—H19	119.1	O41—C41—H41D	109.9
C19—C20—C15	117.84 (12)	C42'—C41—H41D	108.0
C19—C20—C21	118.23 (12)	H41C—C41—H41D	108.3
C15—C20—C21	123.86 (12)	C41—C42—H42A	109.5
N25—C21—C20	111.20 (10)	C41—C42—H42B	109.5
N25—C21—C22	106.42 (10)	C41—C42—H42C	109.5
C20—C21—C22	116.09 (11)	C41—C42'—H42D	109.5
N25—C21—H21	107.6	C41—C42'—H42E	109.5
C20—C21—H21	107.6	H42D—C42'—H42E	109.5
C22—C21—H21	107.6	C41—C42'—H42F	109.5
C26—C22—C21	115.15 (11)	H42D—C42'—H42F	109.5
C26—C22—C23	111.20 (10)	H42E—C42'—H42F	109.5
C21—C22—C23	108.66 (10)	O43—C43—O44	123.79 (13)
C26—C22—H22	107.2	O43—C43—C38	124.62 (12)
C21—C22—H22	107.2	O44—C43—C38	111.50 (11)
C23—C22—H22	107.2	C43—O44—C44	116.36 (12)
O23—C23—C24	109.46 (11)	O44—C44—H44A	109.5
O23—C23—C22	112.17 (10)	O44—C44—H44B	109.5
C24—C23—C22	109.04 (10)	H44A—C44—H44B	109.5
O23—C23—H23	108.7	O44—C44—H44C	109.5
C24—C23—H23	108.7	H44A—C44—H44C	109.5
C22—C23—H23	108.7	H44B—C44—H44C	109.5
			
N25—C1—C2—C3	−120.41 (14)	C2—C1—C24—C32	−54.16 (14)
C24—C1—C2—C3	118.02 (14)	N25—C1—C24—C23	54.20 (13)
N25—C1—C2—C7	59.18 (17)	C2—C1—C24—C23	176.27 (11)
C24—C1—C2—C7	−62.39 (16)	C2—C1—N25—C38	45.07 (14)
C7—C2—C3—C4	0.1 (2)	C24—C1—N25—C38	168.27 (10)
C1—C2—C3—C4	179.73 (14)	C2—C1—N25—C21	177.07 (10)
C2—C3—C4—C5	−0.5 (3)	C24—C1—N25—C21	−59.73 (13)
C3—C4—C5—C6	0.5 (3)	C20—C21—N25—C38	−36.60 (15)
C4—C5—C6—C7	−0.1 (3)	C22—C21—N25—C38	−163.90 (10)
C5—C6—C7—O8	179.55 (16)	C20—C21—N25—C1	−168.13 (11)
C5—C6—C7—C2	−0.3 (2)	C22—C21—N25—C1	64.56 (13)
C3—C2—C7—O8	−179.56 (13)	C21—C22—C26—C27	−150.42 (13)
C1—C2—C7—O8	0.8 (2)	C23—C22—C26—C27	85.44 (15)
C3—C2—C7—C6	0.3 (2)	C21—C22—C26—C31	30.52 (18)
C1—C2—C7—C6	−179.30 (14)	C23—C22—C26—C31	−93.62 (16)
C6—C7—O8—C9	7.0 (2)	C31—C26—C27—C28	−0.1 (2)
C2—C7—O8—C9	−173.11 (13)	C22—C26—C27—C28	−179.20 (14)
C7—O8—C9—C10	172.22 (13)	C26—C27—C28—C29	−1.8 (3)
O8—C9—C10—O11	−70.31 (17)	C27—C28—C29—C30	1.4 (3)
C9—C10—O11—C12	163.33 (13)	C28—C29—C30—C31	0.7 (3)
C10—O11—C12—C13	−157.25 (13)	C27—C26—C31—C30	2.2 (2)
O11—C12—C13—O14	70.49 (16)	C22—C26—C31—C30	−178.71 (15)
C12—C13—O14—C15	−165.89 (12)	C29—C30—C31—C26	−2.6 (3)
C13—O14—C15—C16	−13.83 (19)	C23—C24—C32—C37	−117.39 (15)
C13—O14—C15—C20	165.50 (12)	C1—C24—C32—C37	115.16 (15)
O14—C15—C16—C17	−179.98 (14)	C23—C24—C32—C33	66.03 (18)
C20—C15—C16—C17	0.7 (2)	C1—C24—C32—C33	−61.42 (17)
C15—C16—C17—C18	−0.3 (2)	C37—C32—C33—C34	0.6 (2)
C16—C17—C18—C19	−0.4 (3)	C24—C32—C33—C34	177.24 (14)
C17—C18—C19—C20	0.7 (2)	C32—C33—C34—C35	0.0 (2)
C18—C19—C20—C15	−0.2 (2)	C33—C34—C35—C36	−0.5 (3)
C18—C19—C20—C21	−177.17 (13)	C34—C35—C36—C37	0.4 (3)
O14—C15—C20—C19	−179.83 (12)	C33—C32—C37—C36	−0.7 (2)
C16—C15—C20—C19	−0.48 (19)	C24—C32—C37—C36	−177.45 (16)
O14—C15—C20—C21	−3.06 (18)	C35—C36—C37—C32	0.2 (3)
C16—C15—C20—C21	176.28 (12)	C1—N25—C38—C39	−129.49 (13)
C19—C20—C21—N25	108.80 (13)	C21—N25—C38—C39	99.34 (14)
C15—C20—C21—N25	−67.95 (16)	C1—N25—C38—C43	51.47 (15)
C19—C20—C21—C22	−129.36 (13)	C21—N25—C38—C43	−79.71 (14)
C15—C20—C21—C22	53.89 (17)	N25—C38—C39—C40	−171.04 (13)
N25—C21—C22—C26	171.20 (10)	C43—C38—C39—C40	8.0 (2)
C20—C21—C22—C26	46.87 (15)	C38—C39—C40—O40	−127.60 (17)
N25—C21—C22—C23	−63.33 (12)	C38—C39—C40—O41	55.83 (19)
C20—C21—C22—C23	172.34 (10)	O40—C40—O41—C41	−3.9 (2)
C26—C22—C23—O23	66.75 (14)	C39—C40—O41—C41	172.73 (13)
C21—C22—C23—O23	−61.00 (13)	C40—O41—C41—C42	−171.3 (2)
C26—C22—C23—C24	−171.84 (11)	C40—O41—C41—C42'	−143.9 (5)
C21—C22—C23—C24	60.42 (13)	C39—C38—C43—O43	−146.67 (15)
O23—C23—C24—C32	−59.67 (15)	N25—C38—C43—O43	32.35 (19)
C22—C23—C24—C32	177.29 (11)	C39—C38—C43—O44	36.78 (18)
O23—C23—C24—C1	67.51 (13)	N25—C38—C43—O44	−144.20 (11)
C22—C23—C24—C1	−55.53 (14)	O43—C43—O44—C44	−4.3 (2)
N25—C1—C24—C32	−176.23 (10)	C38—C43—O44—C44	172.24 (14)

### Hydrogen-bond geometry (Å, º)

**Table:**

*D*—H···*A*	*D*—H	H···*A*	*D*···*A*	*D*—H···*A*
O23—H23*O*···O43^i^	0.82 (2)	2.39 (2)	3.1109 (14)	148 (2)

Symmetry code: (i) −*x*+1, −*y*+1, −*z*+1.
